# Complete Genome Sequence of Macrobrachium rosenbergii Golda Virus (MrGV) from China

**DOI:** 10.3390/ani12010027

**Published:** 2021-12-23

**Authors:** Fanzeng Meng, Yiting Wang, Guohao Wang, Tao Hu, La Xu, Kathy F. J. Tang, Weifeng Shi, Fan Zhang, Xuan Dong, Jie Huang

**Affiliations:** 1 College of Fisheries and Life Science, Shanghai Ocean University, Shanghai 201306, China; m190100132@163.com (F.M.); Wgh15866019360@163.com (G.W.); huangjie@ysfri.ac.cn (J.H.); 2 Yellow Sea Fisheries Research Institute, Chinese Academy of Fishery Sciences, Laboratory for Marine Fisheries Science and Food Production Processes, Pilot National Laboratory for Marine Science and Technology (Qingdao), Key Laboratory of Maricultural Organism Disease Control, Ministry of Agriculture and Rural Affairs, Qingdao Key Laboratory of Mariculture Epidemiology and Biosecurity, Qingdao 266071, China; wangyiting022395@163.com (Y.W.); ktangnelson@gmail.com (K.F.J.T.); zhfan1205@163.com (F.Z.); 3 School of Public Health, Shandong First Medical University Shandong Academy of Medical Sciences, Taian 271000, China; uboat@163.com (T.H.); shiwf@ioz.ac.cn (W.S.); 4 Shandong Key Laboratory of Disease Control in Mariculture, Qingdao 266104, China; 18561729912@163.com; 5 College of Marine Science and Biological Engineering, Qingdao University of Science and Technology, Qingdao 266042, China; 6 Network of Aquaculture Centres in Asia-Pacific, Bangkok 10090, Thailand

**Keywords:** *Macrobrachium rosenbergii*, Macrobrachium rosenbergii golda virus, *Goldavirus*, *Nidovirales*, aquaculture

## Abstract

**Simple Summary:**

Macrobrachium rosenbergii golda virus (MrGV) was first identified in *Macrobrachium rosenbergii* in Bangladesh with massive larva death. In this study, the variant of MrGV Mr-18 from China was accidentally found in a meta-transcriptome study of *M. rosenbergii* and compared with MrGV LH1-2018 reported in Bangladesh. Phylogenetic analysis has shown that these two variants belong to the same, yet unclassified, genus. At present, there has no evidence that MrGV Mr-18 causes disease in *M. rosenbergii*. However, we should be alert that MrGV may lead to the mass death of *M. rosenbergii* larvae; thus, surveillance of MrGV in Asia should be given priority.

**Abstract:**

In a meta-transcriptome study of the giant freshwater prawn *Macrobrachium rosenbergii* sampled in 2018 from a hatchery, we identified a variant of Macrobrachium rosenbergii golda virus (MrGV) in postlarvae without clinical signs. The virus belongs to the family *Roniviridae*, and the genome of this MrGV variant, Mr-18, consisted of 28,957 nucleotides, including 4 open reading frames (ORFs): (1) ORF1a, encoding a 3C-like protein (3CLP) (4933 aa); (2) ORF1b, encoding a replicase polyprotein (2877 aa); (3) ORF2, encoding a hypothetical nucleocapsid protein (125 aa); and (4) ORF3, encoding a glycoprotein (1503 aa). ORF1a overlaps with ORF1b with 40 nucleotides, where a −1 ribosomal frameshift with slippage sequence 5′-G^14925^GGUUUU^14931^-3′ produces the pp1ab polyprotein. The genomic sequence of Mr-18 shared 97.80% identity with MrGV LH1-2018 discovered in Bangladesh. The amino acid sequence identities between them were 99.30% (ORF1a), 99.60% (ORF1b), 100.00% (ORF2), and 99.80% (ORF3), respectively. Phylogenetic analysis of the RNA-dependent RNA polymerase (RdRp) proteins revealed that they clustered together and formed a separate cluster from the genus *Okavirus*. The finding of MrGV in China warrants further studies to determine its pathogenicity and prevalence within the region.

## 1. Introduction

*Roniviridae* is a family of the suborder *Ronidovirineae* under the order *Nidovirales*, of which the family name represents rod-shaped nidoviruses referring to the virion morphology of the viruses in the family [1]. Currently, this family includes the genus *Okavirus*, of which three species have been classified: *Gill-associated virus* (GAV, previously known as yellow head virus genotype 2), *Yellow head virus* (YHV, previously known as yellow head virus genotype 1), and *Okavirus 1* (OKV1, previously known as yellow head virus genotype 8) [1,2,3]. Particles in *Roniviridae* are enveloped and rod-shaped (150–200 nm in length, 40–60 nm in diameter) with spike glycoproteins on the surface [4,5,6]. These virions contain a positive-sense single-stranded RNA of 26–27 kb with a 5′-end cap (7-methylguanosine triphosphate) and a polyadenylated 3′-end tail [1,7].

Recently, a new nidovirus named Macrobrachium rosenbergii golda virus (MrGV) LH1-2018 was reported in Bangladesh associated with high mortalities in *M. rosenbergii* hatcheries [8,9]. It shared a nucleotide sequence identity of 46.50% with YHV (EU487200). Compared with YHV, MrGV has a longer genome (29 kb) that encodes four open reading frames (ORFs), including ORF1a, ORF1b, ORF2, and ORF3.

In 2018, we identified a viral sequence from postlarval *M. rosenbergii*, similar to MrGV. We performed reverse transcription polymerase chain reaction (RT-PCR), 5′- and 3′-end rapid-amplification of cDNA ends (RACE) to obtain the full-length genome sequence of this variant and described the molecular and phylogenetic characterizations.

## 2. Materials and Methods

### 2.1. Sample Collection

A batch of *M. rosenbergii* postlarvae (PLs) (mean body length: 0.6 cm) was collected from a hatchery in Jiangsu Province, China, in 2018. There was no obvious death and clinical signs after the PLs were temporarily cultured for one week. The samples were stored in a −80 °C freezer and sent to the Novogene (Beijing, China) through dry ice for sequencing.

### 2.2. RNA Library Construction and Sequencing

Several larvae were ground with liquid nitrogen and mixed for RNA extraction. Total RNA was extracted, and meta-transcriptomic sequencing of the prawn samples was performed as previously described [10]. Using the Epicentre Ribo-zero^TM^ rRNA Removal Kit (Epicentre, Madison, WI, USA) and the Illumina Hiseq platform by Novogene (Beijing, China), ribosomal RNA was removed and then 150-nucleotide (nt) paired-end read sequencing of the RNA libraries was conducted.

### 2.3. Sequence Assembly and Analysis

FASTp is used for quality control of sequencing data to remove adaptors and low-quality reads [11,12]. Sequence assembly and RNA sequence analysis were performed as previously described [10]. After quality control, the sequencing data were de novo assembled using Trinity [13] by BLASTn and BLASTx against the National Center for Biotechnology Information (NCBI) nt database and non-redundant (nr) protein database.

### 2.4. Complete Genome Verification and RCR Detection

To verify the complete genome of MrGV Mr-18, we performed RT-PCR and Sanger sequencing based on contigs as previously described [10]. The 5′- and 3′-terminal sequences of the MrGV Mr-18 genome were determined by rapid amplification of RACE. All primer pairs were provided in Table S1. Total RNA of the prawns was extracted with the UNlQ-10 Column Trizol Total RNA Isolation Kit (Sangon Biotechnology, Shanghai, China) and RevertAid Premium Reverse Transcriptase (Thermo Fisher Scientific, Waltham, MA, USA) was used for reverse transcription. For 5′ RACE, the cDNA was treated with RNase H and the terminal deoxynucleotidyl transferase, followed by a nested PCR and amplicons sequencing. For 3′ RACE, cDNA of MrGV Mr-18 was used as the template and was amplified using the MrGV-specific primer and 3′ adaptor-specific primer. The amplicons were purified for Sanger sequencing.

To further confirm the difference of 5′ untranslated regions (UTRs) between MrGV Mr-18 and LH1-2018, we extracted total RNA from prawn’s tissues (20 mg: containing a mix of gills, hepatopancreas, and muscles) using an RNAprep Pure Tissue Kit (TIANGEN^®^, Beijing, China). The reverse transcription was performed at 42 °Cfor 45 min and 95 °C for 5 min with the random 6 mers and oligo dT using the PrimeScript^TM^ II 1ST Strand cDNA Synthesis Kit (TaKaRa, Dalian, China). PCR was performed in a 25 µL mixture containing 12.5 µL of Premix *Taq* mix (TaKaRa, Dalian, China) (with 0.625 U *Ex Taq*, 5 nmol dNTP, and 50 nmol MgCl_2_), 10 pmol forward/reverse primers (Table S1), and 1 µL of cDNA template. PCR was initiated at 94 °C for 5 min, followed by 30 cycles of 94 °C 30 s, 57 °C 30 s, and 72 °C 30 s, ending at 72 °C for 10 min. Meanwhile, we used the published PCR detection method to detect 13 *M. rosenbergii* [8]. The reverse transcription process was performed as described earlier. PCR amplification was performed in 25 μL reactions using 12.5 µL of Premix *Taq* mix (TaKaRa, Dalian, China) (with 0.625 U *Ex Taq*, 5 nmol dNTP and 50 nmol MgCl_2_), 10 pmol primers (MrGV_F1/R1 in Table S1), and 1 µL of cDNA template. Then, initial denaturation was carried out at 95 °C for 5 min, followed by 30 cycles of 95 °C for 30 s, 58 °C for 30 s and 72 °C for 30 s, ending at 72 °C for 10 min. The size of the product was 319 bp. All amplicons were analyzed and sequenced after 1% agarose gel electrophoresis. Sanger sequencing was completed by Tsingke (Qingdao, China) with an ABI 3730 xl DNA Analyzer (Thermo Fisher Scientific, Waltham, MA, USA)).

### 2.5. Genome Structure and Phylogeny Analysis

ORFfinder (https://www.ncbi.nlm.nih.gov/orffinder/, accessed on 17 December 2021) was used to identify putative ORFs of MrGV Mr-18. The ribosomal shift site was predicted with Fsfinder2 [14]. The conserved protein motifs were identified by Conserved Domain Search (CD-Search) [15], available from GenBank. Pairwise sequence alignment and multiple sequence alignment were performed using the Global Alignment application of European Molecular Biology Laboratory’s European Bioinformatics Institute(EMBL-EBI) with default parameters, which was then manually adjusted using Jalview 2.10.2 [16]. Tmhmm v2.0 [17] was used to identify the transmembrane region of the predicted proteins. ExPASY [18] was used to identify the isoelectric point (PI) and grand average of hydropathicity (GRAVY) with default parameters.

The RNA-dependent RNA polymerase (RdRp) protein sequences of MrGV variants and 24 representative viruses within *Nidovirales* were used to estimate the phylogenetic tree. Two representative viruses from the family *Astroviridae* were used as the outgroup. MEGA 7 was used for multiple sequence alignment and maximum-likelihood phylogenetic analysis [General matrix, a discrete Gamma distribution and a certain fraction of sites are evolutionarily invariable (LG + G + I) evolutionary model] with 1000 bootstrap replications [19].

To better determine the classification of MrGV in *Roniviridae*, MrGV and 8 other viruses of *Roniviridae* with complete genomes were used to build the phylogenetic tree of the RdRp protein sequences. Severe acute respiratory syndrome coronavirus 2 (SARS-CoV-2), a close virus from *Coronaviridae*, was used as an outgroup. MEGA 7 was used for phylogenetic analysis as described above.

## 3. Results

**Figure 1:**
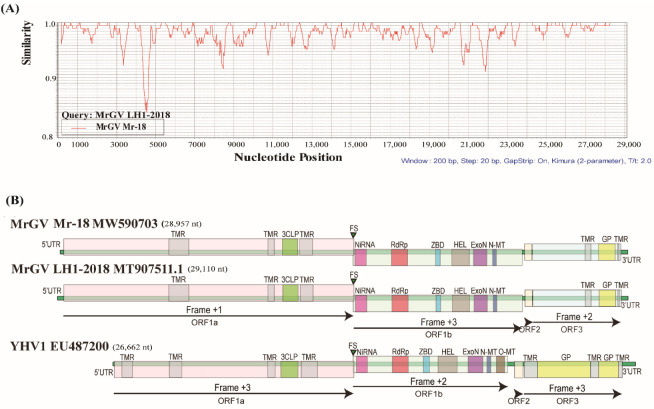
Schematic presentation of the genome structure of MrGV. (**A**) The sequence identity and (**B**) the conserved protein domains of yellow head virus (YHV), Macrobrachium rosenbergii golda virus (MrGV) Mr-18, and MrGV LH1-2018. TMR: Transmembrane region; 3CLP: 3C-like protease; NiRNA: nidovirus RdRp-associated nucleotidyltransferase; RdRp: RNA-dependent RNA polymerase; ZBD: zinc-binding domain; HEL: the helicase; ExoN: 3′–5′ exoribonuclease; N-MT: SAM-dependent N7-methyltransferases; O-MT: 2′-O-methyltransferases; GP: glycoprotein.

We detected 3 positive samples of MrGV from 13 collected *M. rosenbergii* (Figure S1). The genome sequence of MrGV Mr-18 from China was 28,957 nt in length while the genome of LH1-2018 from Bangladesh was 29,110 nt. BLASTn search showed that the nucleic acid sequence identity of the two variants was 98.41% (Figure 1A), with differences mainly in the 5′ UTR. The 5′ UTR of LH1-2018 was longer (333 nt) than that of Mr-18 (168 nt). The complete genome sequence of Mr-18 has been deposited in the GenBank database (accession number: MW590703).

In addition, Mr-18 was predicted to possess four ORFs, including two overlapping ORFs: ORF1a (nt 169–14,970) and ORF1b (nt 14,928–23,561) coding by −1 ribosomal frameshift, and two non-overlapping ORFs: ORF2 (nt 23,684–24,061) and ORF3 (nt 24,086–28,597) (Figure 1B). Using CD-Search and sequence alignment, we identified eight conserved domains, including a Chymotrypsin-like protease (3CLP) (Peptidase_C62, accession number: pfam12380, E-value: 5.17 × 10^−14^) encoded by ORF1a, the nidovirus RdRp-associated nucleotidyltransferase (NiRNA), the RdRp (MERS-CoV-like_RdRp; accession number: cd21592; E-value: 8.49 × 10^−8^), the zinc-binding domain (ZBD_UPF1_nv_SF1_Hel-like; accession number: cd21404; E-value: 1.95 × 10^−8^), the helicase (DEXXQc_Upf1-like; accession number: cd17934; E-value: 2.33 × 10^−^^17^), the exoribonuclease (ExoN) and the guanosine N7-methyltransferase (N-MT) encoded by ORF1b, and the glycoprotein (GP) encoded by ORF3 (Figure 1B) [20]. Three major transmembrane regions (TMRs) were predicted in ORF1a, suggesting a transmembrane protein. There was only a hit of the MrGV LH1-2018 (Identity: 100%; accession number: QOW03297.1) in the BLASTp search using ORF2 as the query; the amino acid sequence identity between MrGV Mr-18 and YHV was only 17.30% in the ORF2 protein, although their GRAVY and PI were very similar (Table S2). ORF3 encoded a putative glycoprotein (Bunya_G1 superfamily; accession number: cl04155; E-value: 1.38 × 10^−10^) and had two TMRs.

One of the features in nidovirus is the presence of a −1 ribosomal shifting site, at which the ribosome shifts from ORF1a to ORF1b and produces a fused ORF1ab polypeptide. Within LH1-2018, the frameshift slippery sequence was 5′-GGGUUUU-3′, followed by a stem-loop stimulatory structure [8]. The same slippery sequence 5′-G^14925^GGUUUU^14931^-3′ and downstream stem-loop structure were found in the ORF1a of Mr-18, and the fused ORF1ab (7797 aa) had a 98.17% nucleotide sequence identity to that of LH1-2018. The ORF2 and ORF3 of Mr-18 had 100.00% and 99.31% nucleotide sequence identity to those of LH1-2018, respectively [8].

**Figure 2:**
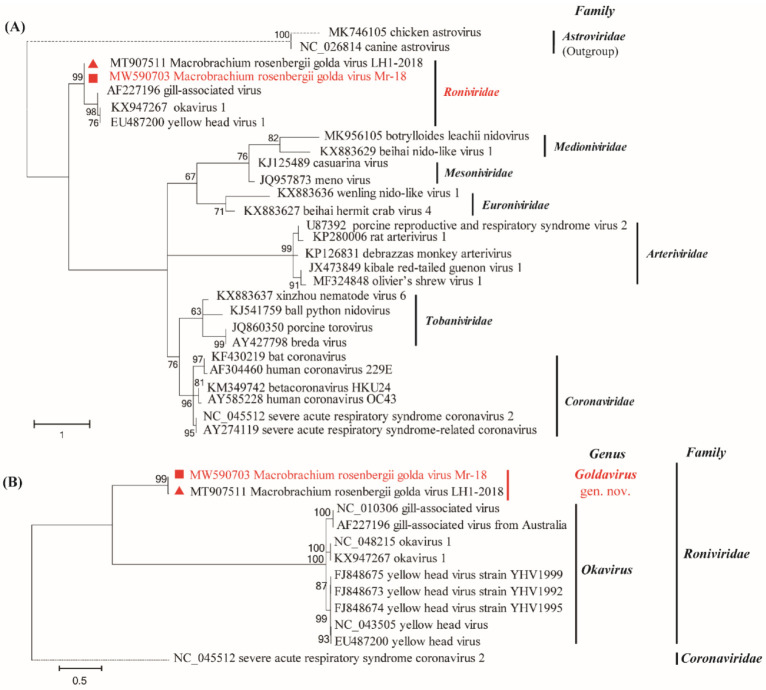
Phylogenetic analysis of the RdRp protein sequences of MrGV Mr-18 and related viruses. (**A**) MrGV Mr-18 and related viruses from the order *Nidovirales*. (**B**) MrGV Mr-18 and related viruses from the family *Roniviridae*. All reference sequences were downloaded from GenBank. Multiple sequence alignment of the RdRp protein sequences was performed using ClustalW, and phylogenetic analysis was constructed using the maximum-likelihood method with the LG + G + I/LG evolutionary model in MEGA 7. In total, 1000 bootstrap re-samplings were run. Bootstrap values ≥ 60% are indicated at the nodes. The MrGV Mr-18 is highlighted with a solid red square.

A maximum-likelihood phylogenetic tree was constructed using the RdRp protein sequences of MrGV and 24 other nidoviruses. The MrGV were placed in the basal position of the family *Roniviridae*, clustered with three okaviruses: YHV, GAV, and OKV1 (Figure 2A), suggesting that MrGV may represent a more ancestral virus of the family *Roniviridae*. The more detailed maximum-likelihood phylogenetic analysis based on the complete amino acid sequence of RdRp of representative variants of *Roniviridae* revealed that the two MrGV variants were grouped together, forming a sister cluster of the genus *Okavirus*. Therefore, the MrGV cluster might represent a new genus of *Roniviridae* (Figure 2B).

## 4. Discussion

MrGV is a novel virus of *Roniviridae*, which has a similar genome structure to okaviruses [21]. However, the ribose 2’-O-methyltransferase (2′-O-MT) was not identified in MrGV, which requires further investigation. The position of ORF2 of MrGV was similar to that of YHV, which may have a nucleic acid binding function encoding the nucleoprotein complexing with the RNA genome to form the nucleocapsid. The PI and GRAVY of ORF2 of MrGV and 13 *Nidovirales* capsid proteins were very similar, suggesting they might have a similar biological function.

Phylogenetic analysis of the RdRp protein sequences showed that Mr-18 belonged to the family *Roniviridae*. We propose that MrGV should be classified as a new genus, provisionally named *Goldavirus*, gen. nov., under the family *Roniviridae*. The relevant Latin name of MrGV is proposed as *Goldavirus macrosenbergii*, gen. nov., sp. nov., based on the binomial nomenclature for virus species [22].

The complete genome of MrGV in farmed *M. rosenbergii* in China provides additional information on the evolution of MrGV and highlights the expanding geographic distribution of this virus. In particular, MrGV has been linked with high mortality in *M. rosenbergii* larvae in Bangladesh [8]. However, MrGV Mr-18 was identified from a giant freshwater prawn postlarvae population without clinical signs in China. Thus, the pathogenicity of MrGV requires further investigation. In 2018, as an important cultured species in the world, the world total output of *M. rosenbergii* was 234.3 kiloton [23]. Given the importance of *M. rosenbergii* culture in Asia, a surveillance plan and biosecurity measures for MrGV should be implemented in this region [24]. Meanwhile, we should pay attention to the detection of MrGV of imported and exported *M. rosenbergii.*

## 5. Conclusions

We reported the complete genome of MrGV Mr-18 from cultured *M. rosenbergii* in China. The nucleotide sequences between MrGV Mr-18 and MrGV LH1-2018 have high identity. MrGV LH1-2018 has caused massive larva death in Bangladesh. Phylogenetic analysis and genomic sequence comparison showed that MrGV Mr-18 is a variant of MrGV LH1-2018.

## Data Availability

All data are available upon request.

## Supplementary Materials

The following are available online at https://www.mdpi.com/article/10.3390/ani12010027/s1, Figure S1. Electrophoretogram of molecular detection of Macrobrachium rosenbergii Goldavirus. Table S1. Primers used for amplification and sequencing of the Macrobrachium rosenbergii golda virus (MrGV) Mr-18. Table S2. Isoelectric point (PI) and grand average of hydropathicity (GRAVY) of replicase polyprotein pp1ab and nucleocapsid protein.
