# Supplementary material for: Complete Genome Sequence of Macrobrachium rosenbergii Golda Virus (MrGV) from China
Source: Animals (Basel). 2021 Dec 23;12(1):27. doi: 10.3390/ani12010027 (PMC8749832; doi:10.3390/ani12010027)
Supplement: Supplementary file 1 [file animals-12-00027-s001.zip › animals-1435824-supplementary.pdf]

Table S1. Primers used for amplification and sequencing of the Macrobrachium rosenbergii golda virus (MrGV) Mr-18.

Primer	Sequence 5'-3'
MrGV_F1	TTTGCCCAGGTTAATTGCCC
MrGV_R1	ACAAGTGCCAGTGAGACGTA
MV-F1	TCAACTGGCCGAGTTTGGC
MV-R1	CGTTTACAAAGATCAACAGGTTGG
MV-F2	GAGGTTATATCGTTCGAGTCTGGTG
MV-R2	GCATGGTGGCCTAGACGAAAC
MV-F3	TCTGCTGATCCCTTTATAACTGGAG
MV-R3	GTAATTGTTCTGGAGCCACATCG
MV-F4	GGTTTTGGCATTGAAACCAGT
MV-R4	ACCTTAGCGAGGAAACTCGAGAA
MV-F5	AATAATGCTTTAACTCCTGGTGCTG
MV-R5	ATCCATATCGTGCGCTAGCATAA
MV-F6	CCTATTGGGTCACCGGCTTA
MV-R6	AAGGAAGGATCCAAACTCACCC
MV-F7	AGAGGCATATGGTATCATGGAAAG
MV-R7	CAGTAAAACCGACCAAATAACCAC
MV-F8	GAGACTGTTCTTGAGCTCCTACCTT
MV-R8	AATGCAAATGAGTCATCAGTCCAG
MV-F9	CCTGCAGCTCAAGGGTGCTA
MV-R9	GCATAGGCGTTAGCATCTGTAACA
MV-F10	GTTCAGATACAACCATCTCACCAGA
MV-R10	CACAAAAGGAGCGATCCTTTGT
MV-F11	CTCTCGTCTGTAATGCAGTCAGC
MV-R11	ATAACCTTGATCTCCGGTTATACG
MV-F12	CAGCGTTGATGATGTTGTTGTTG
MV-R12	AATCCTGCCAATGCCAAATG
MV-F13	GCTCTAGTCAGAGACAATTGGGTTA
MV-R13	CACAAGTAACTACAGCTTCATCCGT
MV-F14	AACTCCGTCACTGATGAAGCTATT
MV-R14	GAACACGTTGCCTAGAGCAATCT
MV-F15	TTCATAATGTTGTCGAATGCCC
MV-R15	GCTTTCATGTAAAGACAGTTGCG
MV-F16	TTACCGAGAGGTTTCTTCTATGAGC
MV-R16	GAGGATGCCATCATAAGGTAGAAGT
MV-F17	CAATGTACAGCCTGTCGGGG
MV-R17	AGGTCTAAAAGCACCATAAGCCTTA
MV-F18	TCGTGGTGTTTCTTCAGGTGAT
MV-R18	GCGAAACATTCCACCTCATTATC
MV-F19	GTTGAGTTGAGCCGGATTTTTC
MV-R19	ACTGCATGTAAAATCCCGGC

MV-F20	GCAATGCACAAATATGGGTTTC
MV-R20	GTTGCATAGGATCACCAACCAA
MV-F21	GGTGGGCTTGTTTTTGCCT
MV-R21	CAGTCATAGAGAAACGCCATATCC
MV-F22	GCGGCCAGCGTATAAGCA
MV-R22	TCCACAGACGGAAGACCTTAGAA
MV-F23	TTTATGGTCAATGTGGCTCAGTTC
MV-R23	TCCGTTATAATAGGACGAATGGTAC
MV-F24	GCACTCTTATCCTGGCTTAGTTGTC
MV-R24	ACCTGTCATAATAACAGACCCGC
MV-F25	TGTGGGTTTTTGTGATATCACAGTC
MV-R25	CATATCACTTCACAAGGCGCAG
MV-F26	ATTGCCTTTGCAAATATAGCGAG
MV-R26	CACCGGGTACACCATTTGTTT
MV-F27	ACAATGGTTATGGTCTCGTTGC
MV-R27	TATCAACAACCTTGACGACAACG
MV-F28	GGAATAATGGCGAACTTGAGG
MV-R28	CACCCACAGTAACCTCTATTTCGT
5′adaptor	GCTGTCAACGATACGCTACGTAACGGCATGACAGTGG GIIGGGIIGGGIIG
3′adaptor	GCTGTCAACGATACGCTACGTAACGGCATGACAGTGT TTTTTTTTTTTTTTTTT
5.3′outer	GCTGTCAACGATACGCTACGTAAC
5.3′inner	GCTACGTAACGGCATGACAGTG
MrGV LH1-2018-F1	GTATGAGCACAGTAAGCAAGC
MrGV CN-18-R1	GTATGAACACAGTAAGCAAGC
MrGV LH1-2018-F2	CCAGACACCAAAGGATCTTAAC
MrGV CN-18-R2	GTATGAACACAGTAAGCAAGC
MrGV CN-18-F3	TCAGTTGCCGTGACTTTACTCC
MrGV CN-18-R3	ACAGCATCAGTGGTATGAACAC

Table S2. Isoelectric point (PI) and grand average of hydropathicity (GRAVY) of replicase polyprotein pp1ab and nucleocapsid protein.

Accession number	Name	pp1ab		nucleocapsid protein	
		pI	GRAVY	pI	GRAVY
MW590703	MrGV Mr-18	6.38	0.089	9.88	-0.800
MT907511	MrGV LH1-2018	6.37	0.089	9.88	-0.800
AF227196	GAV	7.04	-0.228	9.84	-0.759
NC_048215	OKV1	7.00	-0.257	9.92	-0.718
EU487200	YHV	7.03	-0.234	9.98	-0.726
NC_045512	SARS-CoV-2	6.34	-0.070	10.07	-0.971
AY274119	SARS-CoV	6.20	-0.071	10.11	-1.027
AY585228	HCoV_OC43	6.64	0.065	9.65	-0.896
KM349742	BetaCV HKU24	6.64	0.070	9.72	-0.862

AF304460	HCoV_229E	6.51	0.049	9.72	-0.922
KF430219	BtCoV CDPHE15	6.52	0.040	9.80	-0.888
AY427798	BRV	6.15	-0.004	11.82	-0.960
JQ860350	POTV	6.03	-0.036	12.10	-0.848
DQ898157	WBV	6.50	-0.160	9.82	-0.815
KJ541759	BPNV	7.77	-0.562	9.99	-0.844

Abbreviations: MrGV Mr-18: macrobrachium rosenbergii golda virus Mr-18; MrGV LH1-2018: macrobrachium rosenbergii golda virus LH1-2018; GAV: gill-associated virus; OKV1: okavirus 1; YHV: yellow head virus; SARS-CoV-2: severe acute respiratory syndrome coronavirus 2; SARS-CoV: severe acute respiratory syndrome-related coronavirus; HCoV_OC43: human coronavirus OC43; BetaCV HKU24: betacoronavirus HKU24; HCoV_229E: human coronavirus 229E; BtCoV CDPHE15: bat coronavirus CDPHE15; BRV: breda virus; PoTV: porcine torovirus; WBV: white bream virus; BPNV: ball python nidovirus.

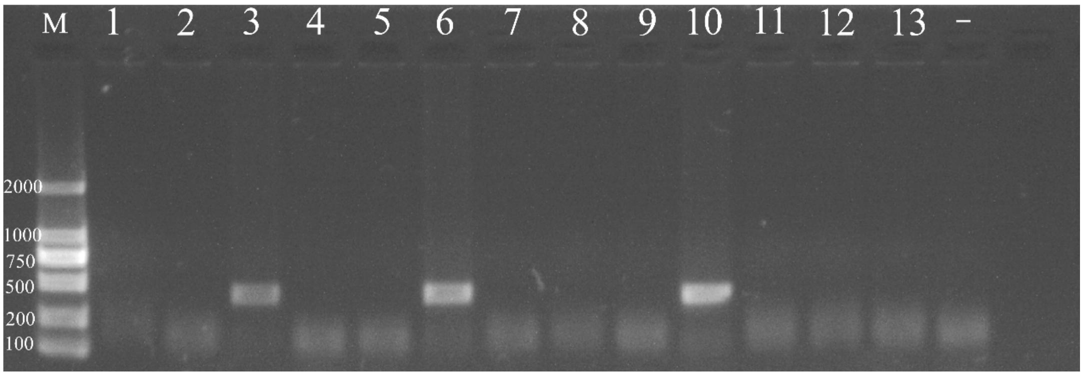

Figure S1. Electrophoretogram of molecular detection of *Macrobrachium rosenbergii Goldavirus.* M: 2 kb marker DNA. -: Water was used as blank control. 1-13: Sample names.
